# Using Patient-Held Devices to Measure Variations in Resting Heart Rate and Step Count Prior to Presentation With an Acute Illness: International, Multicenter Flash Mob Feasibility Study

**DOI:** 10.2196/76218

**Published:** 2025-12-15

**Authors:** Jason G A den Duijn, Ahmed A M Hajjaj, John Kellett, Erika Frischknecht Christensen, Harm R Haak, Mikkel Brabrand, Christian H Nickel, Prabath W B Nanayakkara, Christian P Subbe, Jelmer Alsma, Safer@Home, Anne Lund Krarup, Research Consoritum Acute Medicine (ORCA), Suzanne Schol-Gelok

**Affiliations:** 1 Department of Medical Oncology, Erasmus University Medical Center, Dr. Molewaterplein 40, Rotterdam, 3015 GD, The Netherlands, 0031 655014806; 2 Department of Acute Medicine, Ysbyty Gwynedd Hospital National Health Service Trust, Bangor, United Kingdom; 3 School of Clinical and Biomedical Sciences, University of Greater Manchester, Bolton, United Kingdom; 4 Center for Prehospital and Emergency Research, Department of Emergency Medicine and Trauma Center, Aalborg Hospital and Aalborg University, Aalborg, Denmark; 5 Department of Internal Medicine, Máxima Medisch Centrum, Veldhoven, The Netherlands; 6 Department of Health Services Research, Aging and Long Term Care, Care and Public Health Research Institute School for Public Health and Primary Care, Maastricht, The Netherlands; 7 Department of Emergency Medicine, Odense University Hospital, Odense, Denmark; 8 Emergency Department, University Hospital Basel, University of Basel, Basel, Switzerland; 9 Section of Acute Medicine, Department of Internal Medicine, Amsterdam Public Health Research Institute, Amsterdam University Medical Center Location Vrije Universiteit Amsterdam, , Amsterdam, The Netherlands; 10 North Wales Medical School, Bangor University, Bangor, The Netherlands; 11 Department of Internal Medicine, Erasmus University Medical Center, Rotterdam, The Netherlands; 12 See Acknowledgments

**Keywords:** smart device, acute care, flash mob, vital signs, monitoring

## Abstract

**Background:**

Many patients experience a gradual decline in health before seeking hospital care, with subtle changes in vital signs such as increased heart rate or decreased mobility. Recognizing deviations from baseline vital signs can support clinical decision-making, especially admission decisions. Smart devices (ie, smartphones, smartwatches, and activity trackers) track health metrics like heart rate and step count, offering new opportunities to estimate illness severity and track deterioration early.

**Objective:**

This study aimed to assess the feasibility of using heart rate and step count measurements from smart devices (ie, smartphones, smartwatches, and activity trackers) to enhance the evaluation of patients presenting with acute illness in emergency settings.

**Methods:**

We conducted an international multicenter prospective observational study using the flash mob study design in 34 hospitals in the Netherlands (n=17), the United Kingdom (n=7), Denmark (n=9), and Switzerland (n=1) in May 2024. Researchers collaborated with patients to complete questionnaires upon an acute care (ie, emergency department, acute medical unit, same day emergency care) visit and extracted physiological data from their smart devices.

**Results:**

Among patients with an acute care visit (n=1137), 40% (n=452) had a smart device with health data. These patients tended to be from a higher educational level and in relatively good health. Only half had retrievable heart rate or step count data, resulting in a usable data set for 20% (n=209) of the total study population. Analysis showed a significant increase in heart rate (*P*<.001) and a decrease in step count (*P*<.001) in the days preceding their hospital visit. Both heart rate (*P*=.04) and step count (*P*=.04) on the day before presentation were significantly associated with disposition.

**Conclusions:**

Our study demonstrates the feasibility of using a patient’s personal smart device to monitor vital signs in the days leading up to an acute care visit. In a selected patient group, significant changes in heart rate and step count were observed prior to hospital presentation, suggesting that disposition may be predicted using data collected from the patient’s own device. High-risk patient groups, who might benefit the most from digital health monitoring, are currently underrepresented among device users.

## Introduction

Many patients presenting to hospitals with acute medical complaints experience a gradual decline in health over days or even weeks before seeking care [1]. During this period, subtle yet significant changes in vital signs, such as an increased heart rate or decreased step count, may already be present, signaling underlying physiological stress [2-4]. In the emergency department (ED), acute medical unit (AMU), or same-day emergency care (SDEC), the decision to admit a patient to the hospital is influenced by various factors, including patient symptoms, vital signs, past medical history, medication use, diagnosis, as well as availability of social support at home [5-7], and the estimated risk of clinical deterioration in the subsequent hours and days [8]. Assessing how far a patient’s vital signs deviate from their baseline may provide valuable insight to support admission decisions.

Reference values from healthy individuals, recorded during periods of physiological stability, are often used for this purpose [9]. The extent of deviation from physiological baseline, as well as the number of vital signs affected, correlates positively with the frequency of adverse events and is widely recognized as a measure of illness severity [9]. These above or below average “abnormalities” can be assessed with generic tools such as the National Early Warning Score (NEWS) [10] or with disease-specific tools such as the CURB65 for pneumonia [11] and the Blatchford score for bleeding in the upper gastrointestinal tract [12]. These scoring systems are based on population data and may under- or overestimate risk in individual patients. The vital signs of a healthy individual have a natural variability influenced by several factors, including age, sex, body composition, medication use, genetics, and physical condition [13-15]; very fit individuals, for example, often have very low heart rates [16]. Therefore, personal baseline measurements may offer a more tailored approach to evaluating deviations than population averages to determine the illness severity of an individual patient [17].

The use of smart devices is growing rapidly, with reports of 76% to 97% of the population in the United States [18] and 82% to 98% in the United Kingdom [19] owning smartphones, with rates varying between age groups. Many smart devices (ie, smartphones, smartwatches, and activity trackers) measure basic health metrics, including heart rate and step count. These may provide an opportunity for patients to track and share their baseline values over time [20], potentially enabling a more accurate estimation of their illness severity [1] and its rate of deterioration [21]. This has already been demonstrated for several medical conditions, for example, new or paroxysmal atrial fibrillation and COVID-19 [22-27].

The aim of this study was to assess the feasibility of using heart rate and step count measurements captured by smart devices (ie, smartphones, smartwatches, and activity trackers) to enhance the assessment of patients presenting as emergencies with an acute illness. Feasibility was defined as having a smart device with heart rate or step count data recorded at least twice between 30 days (baseline) before and on the day of admission, enabling trend analysis, and measured as the percentage of patients with an acute care visit (ED, AMU, or SDEC).

## Methods

### Study Design and Setting

We conducted an international, multicenter, prospective observational study using the flash mob study design [28] across 34 hospitals in four countries in May 2024: the Netherlands (n=17), the United Kingdom (n=7), Denmark (n=9), and Switzerland (n=1). This study was previously described in a protocol paper [29]. Each site recruited patients over a single day between 8 AM and 10 PM.

### Ethical Considerations

Ethical approval was obtained from the Medical Ethical Assessment Committee (MECC-2022-0795) of the Erasmus University Medical Center, Rotterdam, the Netherlands, and the London–Harrow Research Ethics Committee, Bristol, United Kingdom (IRAS 321129). Approval for all other study sites was acquired in accordance with national and local regulations. All patients gave written informed consent to participate in the study. All collected data were pseudonymized before data analysis.

### Participants

All patients presenting to ED, AMU, or SDEC were screened for inclusion and exclusion criteria and asked about their use of monitoring devices. Inclusion criteria were ≥18 years, having a device (smartphone or smartwatch) capable of measuring resting heart rate or step count, and the ability to provide informed consent. Patients were excluded for presentation with trauma or clinical instability, as determined by the treating physician. Written informed consent was obtained by trained investigators. Researchers collaborated with patients to complete questionnaires, after which physiological data were extracted from the patients’ devices.

### Variables

Patient variables included gender, age groups (18‐30, 31‐50, 51‐65, and 65+ years), educational attainment, digital literacy, and device brand and type. The resting, maximum, and minimum heart rates were collected from the patient’s device for 4 time points: 30 days, 7 days, 1 day before the hospital visit, and on the day of admission. In addition, the heart rate measured at presentation to hospital was extracted from the patient’s clinical record. Step count data were collected from the patient’s device for 9 time periods: 30 days, the preceding 7 days before the hospital visit, and the day of the visit. As changes in step count fluctuate more, we chose to collect these on more time points than heart rate. For either heart rate or step count, a minimum of two time points had to be available for data analysis. Additionally, the value of the NEWS, the Clinical Frailty Scale (CFS) [30], and a standardized assessment of gait were recorded. Follow-up data collected up to 7 days after presentation included the date of visit, disposition (admission to hospital or discharge), admission to intensive care or high care areas, death, and discharge, if applicable. Patients were followed up over a period of up to 7 days.

### Study Size

Due to the lack of published data on the frequency distribution of the relevant parameters in the hospitalized population, a formal sample size calculation was not feasible. We estimated that around 200 patients with smart devices and the corresponding data would be recruited based on the number of sites, the average amount of patients visiting the ED daily, and the percentage of people with a smart device [18,19,31]. This cohort size was considered representative in providing data with high external validity.

### Statistical Methods

The primary outcome was feasibility, which was defined as having a smart device with heart rate or step count data recorded on at least twice between 30 days (baseline) before and on the day of admission, enabling trend analysis, and measured as the percentage of patients with an acute care visit. Patient characteristics were reported as absolute numbers per category or subcategory for the total patient population, discharged patients, and admitted patients. Variables were assessed for normal distribution. The heart rate and step count data were compared for their respective mean or median for the different time points. Heart rate changes were also assessed relative to baseline. Both were tested for significant interaction between time, disposition, and change in either heart rate or step count using a generalized linear model (GLM). To further assess the effect of time on change in heart rate and step count, a generalized linear mixed model (GLMM) with binomial distribution and random effects for country and hospital was conducted. Data were collected and safely stored using Castor EDC [32] and analyzed using SPSS (version 28, IBM Statistics). Statistical significance was defined at *α*=.05.

## Results

### Feasibility

**Figure 1.**
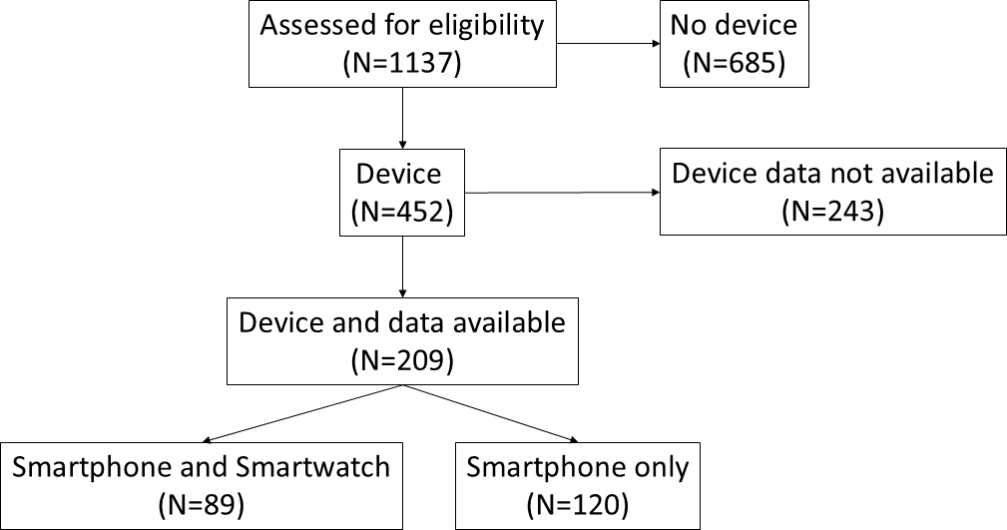
Flowchart of the included patients.

A total of 1137 patient cases were screened for this study (Figure 1). Of these, 685 were excluded for the following reasons: not having a smart device, not having their device with them during presentation to the hospital, not measuring health data with the smart device, and not having worn their device in the previous days due to illness. A total of 243 patients were excluded because of having insufficient amount of data, and 209 (18%) patients formed the core group for further analysis. Among these patients, 89 (43%) used a smartwatch (in combination with a smartphone) and 120 (57%) only used their smartphone (Multimedia Appendix 1). Heart rate data were recorded in 84 (40%) patients, and step count data were available in 207 (99%) patients.

### Characteristics of Subjects

We included a total of 209 patients who had either a smartwatch or smartphone that measured heart rate or step count in the week prior to their visit (Table 1). Distribution between male and female patients was equal. Most included patients were aged under 65 years, had attained higher education, and were confident in the usage of internet and applications. The overwhelming majority of the patients had low NEWS scores, low frailty scores, and normal gait. In terms of patient disposition, 75 (36%) patients were admitted and 126 (60%) were discharged, and for 8 (4%) patients, the outcome was unknown. Follow-up data showed that no patients died, 3 were admitted to the intensive care unit, and 122 were discharged within 7 days.

**Table 1.** Characteristics of included patients.

Characteristics	All (n=209), n (%)	Admitted (n=75, 36%), n (%)	Discharged (n=126, 60%), n (%)
Gender			
Female	109 (52)	41 (55)	65 (52)
Male	100 (48)	34 (45)	61 (48)
Age group			
18‐30 y	37 (18)	14 (19)	23 (18)
31‐50 y	54 (26)	14 (19)	38 (30)
51‐65 y	65 (31)	25 (33)	37 (29)
65+ y	53 (25)	22 (29)	28 (22)
Educational attainment			
Primary school	15 (7)	7 (9)	7 (6)
Secondary school	68 (33)	24 (32)	39 (31)
Higher education	124 (59)	42 (56)	80 (63)
Unknown	2 (1)	2 (3)	0 (0)
Confidence in internet usage			
Very	125 (59)	41 (55)	80 (63)
Fairly	65 (31)	27 (36)	35 (28)
Unsure	10 (5)	2 (3)	7 (6)
Not very	6 (3)	3 (4)	3 (2)
Not at all	3 (1)	2 (3)	1 (1)
Confidence in app usage			
Very	131 (63)	43 (57)	85 (67)
Fairly	59 (28)	24 (32)	30 (24)
Unsure	10 (5)	4 (5)	6 (5)
Not very	6 (3)	3 (4)	3 (2)
Not at all	2 (1)	1 (1)	1 (1)
Unknown	1 (0)	0 (0)	1 (1)
National early warning score			
Low (0‐2)	178 (85)	57 (76)	115 (91)
High (3+)	27 (13)	18 (24)	8 (6)
Unknown	4 (2)	0 (0)	3 (2)
Clinical Frailty Scale			
1-2	152 (73)	44 (59)	104 (83)
3+	57 (27)	31 (41)	22 (17)
Normal gait			
Yes	187 (89)	63 (84)	117 (93)
No	17 (8)	9 (12)	7 (6)
Unknown	5 (2)	3 (4)	2 (2)

### Change in Heart Rate and Step Count

**Figure 2.**
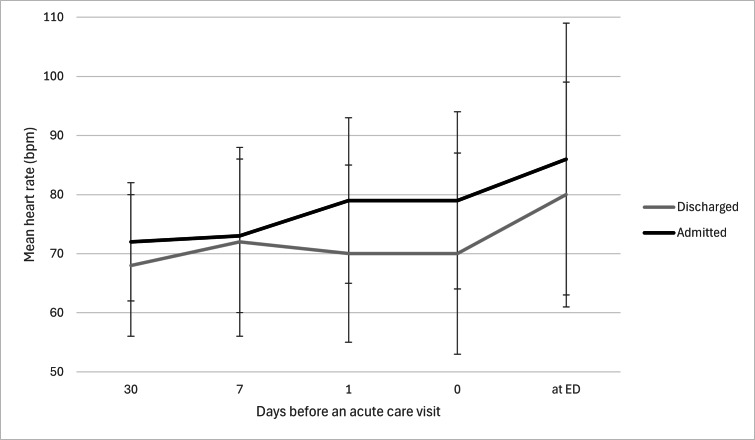
Change in mean heart rate in days before an acute care visit. bpm: beats per minute; ED: emergency department.

**Figure 3.**
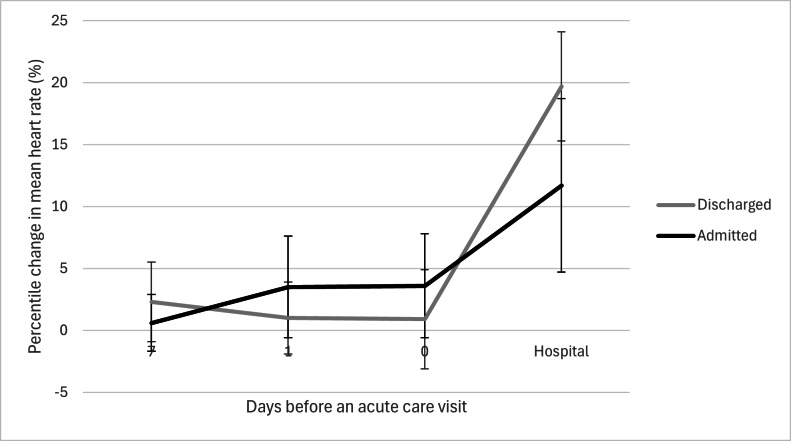
Percentile change in mean heart rate in days before an acute care visit.

The mean resting heart rate increased over the 30 days preceding hospital attendance in all patient groups, including both admitted and discharged patients (Figure 2, Multimedia Appendix 2). Patients who were admitted had a higher mean resting heart rate than those who were discharged. The GLM showed a significant change in the mean resting heart rate over time (*P*<.001), but there was no significant interaction between time and patient disposition (*P*=.23). The GLMM correctly classified 85% (51/60) of cases, with higher accuracy for discharge (38/40, 95%) than for admission (13/20, 65%). Among the predictors, only the mean resting heart rate on the day prior to presentation was significantly associated with patient disposition (β=–.083, SE=.038, *P*=.035). A higher resting heart rate on the day before an acute care visit was linked to a lower likelihood of admission (odds ratio 0.92 per beats per minute [bpm], 95% CI 0.85‐0.99). Mean resting heart rate at 1 month (*P*=.66), 1 week (*P*=.23), on the day of presentation (*P*>.99), and during admission (*P*=.92) was not significantly associated with disposition. The percentage increase in the mean resting heart rate from baseline prior to hospital admittance was greater in admitted patients than in those who were discharged (Figure 3). In contrast, patients discharged after an acute care visit exhibited the highest percentile change in the mean resting heart rate.

**Figure 4.**
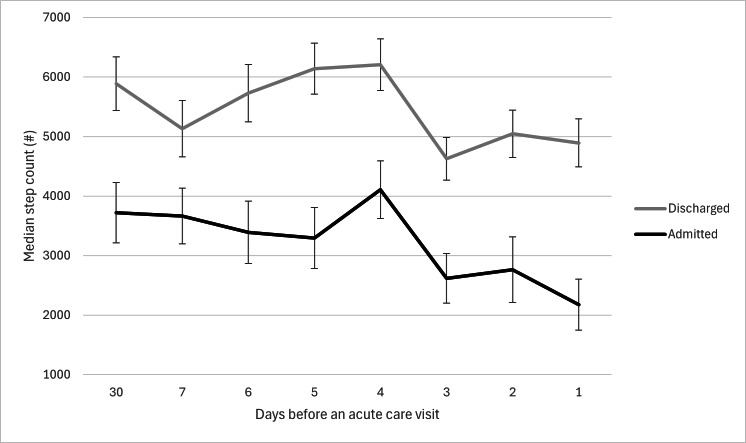
Change in median step count in days before an acute care visit.

The median step count decreased over the 30 days leading up to hospital presentation in both patient groups (Figure 4, Multimedia Appendix 3). The GLM showed a significant change in median step count over time (*P*<.001) but no significant interaction between time and disposition (*P*=.10). The GLMM correctly classified 82% (112/136) of cases, with higher accuracy for discharge (83/88, 94%) than admission (29/48, 60%) patients. Among the predictors, only median step count on the day prior to presentation was significantly associated with disposition (β≈.0001, SE≈0.00009, *P*=.04). A lower median step count on the day before hospital presentation predicted an increased likelihood of admission (odds ratio=0.90 per 1000 steps, 95% CI 0.82‐1.00). Median step counts at 30 (*P*=.43), 7 (*P*=.78), 6 (*P*=.74), 5 (*P*=.84), 4 (*P*=.22), 3 (*P*=.11), and 2 days (*P*=.18) prior to hospital presentation were not significantly associated with disposition.

## Discussion

### Key Results

This study found that 40% of patients with an acute care visit had a smart device. In only 50% of the included patients, heart rate and step count data were available for trend analysis, resulting in data available in 20% of the total cohort. Within these patients, a significant increase in heart rate and a decrease in step count were observed several days before they sought emergency care.

### Interpretation 

#### Feasibility

Feasibility was defined as having a smart device with heart rate or step count data recorded at least twice between 30 days (baseline) before and on the day of admission, enabling trend analysis, and measured as the percentage of patients with an acute care visit (ED, AMU, or SDEC). Device ownership, and thus feasibility, was lower in our study compared to reported daily use in the community (smartphone, 18% vs 90%; smartwatch, 8% vs 49%) [18,33-35]. We attribute this difference to two causes. First, the ED population is not representative of the general population. Patients who are at higher risk of requiring acute care are often older and from lower socioeconomic backgrounds [36,37], which contributes to lower device ownership [35]. Second, 40% had a smart device that measured heart rate and 99% had one that measured step count, leaving part of the potential value of health monitoring apps unused. This is more commonly seen in older patients [38]. Additionally, we excluded patients who did not have their smart device with them or had insufficient data (54%). Both were categorized as having no smart device. Nevertheless, in patients who used their smart device, the results demonstrated the potential clinical value of smart device recording of heart rate and step count data for assessing disease severity and rate of deterioration. These findings show that society is still a long way from converting care from analog to digital, but that the trend is slowly setting in.

#### Change in Heart Rate and Step Count

Changes in both step count and heart rate are associated with clinical deterioration [39,40]. In our study, we found that variations in these metrics on the day prior to an acute care visit significantly predicted patient disposition.

Interestingly, in our population, a greater increase in resting heart rate was associated with a lower likelihood of hospital admission. We hypothesize that this counterintuitive finding may be explained by the pattern of percentile change in resting heart rate (Figure 3). Discharged patients tended to exhibit a brief, sharp increase, whereas admitted patients showed a more gradual and prolonged rise. This difference may reflect variations in autonomic regulation: healthier individuals—those ultimately discharged—could have greater heart rate variability (HRV), allowing for more pronounced fluctuations. Although HRV was not directly measured in our study, literature suggests that higher HRV is associated with better health status and resilience to stressors, potentially explaining the observed spike in heart rate in discharged patients [41,42]. Though, it is important to note that the group of discharged patients maintained a lower overall mean resting heart rate compared to those who were admitted.

Other studies have similarly demonstrated that changes in heart rate are linked to illness severity and that failure to normalize heart rate by the time of disposition is associated with worse outcomes [1,41-45]. Furthermore, the mean resting heart rate increased in both groups, although it remained within the normal range defined by early warning scores such as NEWS.

Notably, heart rate measured by the patient’s device on the day of the acute care visit and in the hospital during triage (ED, AMU, or SDEC) showed an increase in both discharged (from 75 to 82 bpm) and admitted (from 79 to 88 bpm) patients. This increase begins on day −1 and appears predictive of admission. The rise may reflect acute physiological stress due to illness, emotional stress associated with hospital visits, or differences in measurement context. While discrepancies between consumer-grade devices and hospital equipment could be a factor, prior validation studies suggest that smartwatches and activity trackers provide sufficiently accurate measurements [46,47], making this explanation less likely.

The overall difference between admitted and discharged patients highlights a substantial disparity in general physical fitness between the 2 groups. Compared to discharged patients, those who were admitted had a significantly lower overall median step count, with a further decline beginning 2 days prior to the acute care visit. This pattern may enable the identification of patients at risk of admission as early as a day before presentation. These findings support previous findings that physical activity, including step count, tends to decline as patients become more acutely ill [3,48,49].

These findings underscore the importance of using individualized baselines and relative changes as reference points, highlighting the potential of personalized medicine.

#### Study Population

Our final study population was reflective of the general acute care population for both age and gender but reported a higher level of education [50]. Higher education usually corresponds with a higher socioeconomic class, and a higher socioeconomic class has a lowered risk of acute care visits and poor health outcomes [36,37]. Furthermore, our population was confident in the usage of both the internet and applications, indicating strong digital health literacy [51]. This is most likely because a higher education is associated with a higher digital health literacy [52]. The included patients had mostly low NEWS, CFS, and normal gait. This indicates that our participants were relatively healthy compared to the average acute care population. This is supported by a recent European study showing that 40% of older people using the emergency care had CFS 5+ [53]. Furthermore, many patients these days visit the ED for acute complaints of chronic diseases, demonstrating the increasing fragility of the ED population [54]. Based on our findings, it seems the patients who would potentially benefit the most from digital health monitoring do not use it, as opposed to the relatively healthy patients who already use it. In summary, we included a group of patients from a high socioeconomic class in relatively good health, which is not consistent with the general population of acute patients.

### Strengths and Limitations

The major strength is the generalizability of this point prevalence study into the use of smart devices in Northwestern Europe, including several countries, regions, and hospital settings. Additionally, our study highlights the clinical value of patient-held sensors and understanding patients’ own reference value. This could be used to track a patient’s health remotely and proactively (ie, monitoring individuals before they have an acute illness and become patients) by both patients and health care providers.

Our study had several limitations. First, by only including patients who had a smart device and sufficient data, we created a selection bias. We selected patients with higher education and most likely a higher socioeconomic status, representing a healthier population. However, we expect the underlying pathophysiology to be unaffected by this selection. Second, because of the flash mob research design and because we wanted to minimize the workload for the participating centers, we chose to collect only a limited number of parameters. Resting heart rate between 6 and 2 days before an acute care visit, reason for attendance, medication use, medical history, psychosocial aspects, and ethnicity were not considered even though these parameters could have provided more insight into which groups of patients are being admitted. Finally, the sample size was relatively small, making the subgroup analysis (eg, device type) not feasible. The small sample size and selection bias toward individuals with less compromised physiology may lead to the underestimation of the changes before and on the day of contact with the emergency services.

### Future Perspectives

Proactive monitoring of health data in populations at high risk of acute illness could enable earlier identification of deteriorating health. Monitoring on a daily basis may help detect subtle changes in vital signs or physical activity, allowing for timely medical assessment in primary or ambulatory care settings and potentially preventing a hospital visit. This strategy is already recommended for atrial fibrillation detection by the European Society of Cardiology guidelines 2024 [55]. This benefit is potentially greatest for frail patients, but they are less likely to use a smart device to monitor their health. In addition, changes in vital signs may be more subtle in frail patients, making it more difficult to detect changes. In fitter patients, changes in heart rate and step count may be steeper, but as shown in our study population, the theoretical benefit in a nonselected population is limited. Overall, patients might be encouraged to bring their smart devices with them to an acute care visit, and clinicians might consider asking patients to look at these smart devices, as information on recent heart rate and step counts may be helpful, which can be used as a valid measurement of resting heart rate, as shown by several studies [46,47]. Additionally, both users and manufacturers of smart devices can help health care providers by expanding their reach. Currently, users tend to be younger, more educated, and digitally literate patients, while devices could show clinically valuable trends for all. Both patients and professionals should be wary of misdiagnosis or overdiagnosis. This study provides a foundation for further research to help patients and clinicians pick up early deterioration in health, which might attract interest from tech companies and mobile application developers. Furthermore, a targeted information campaign could be considered, which should be aimed at high-risk patient groups.

### Conclusions

Our study showed that it is feasible to use a patient’s own smart device to measure vital signs in the days preceding an acute care visit. We found a significant change in heart rate and step count prior to presentation to hospital, where disposition can be predicted using a patient’s own smart device data. These smart devices are mostly used by younger and healthier patients with higher educational attainment. The use of a patient’s own smart devices for health monitoring in high-risk patient groups is very limited.

## Supplementary material

10.2196/76218 Multimedia Appendix 1 Characteristics of devices of included patients.

10.2196/76218 Multimedia Appendix 2 Change in heart rate in patients.

10.2196/76218 Multimedia Appendix 3 Change in step counts in patients.

10.2196/76218 Multimedia Appendix 4 Contributing author list.[Aff aff1]

## Abbreviations

AMU: acute medical unit
bpm: beats per minute
CFS: Clinical Frailty Scale
ED: emergency department
GLM: generalized linear model
GLMM: generalized linear mixed model
HRV: heart rate variability
NEWS: National Early Warning Score
SDEC: same day emergency care
