# Supplementary material for: Using Patient-Held Devices to Measure Variations in Resting Heart Rate and Step Count Prior to Presentation With an Acute Illness: International, Multicenter Flash Mob Feasibility Study
Source: JMIR Cardio. 2025 Dec 15;9:e76218. doi: 10.2196/76218 (PMC12705129; doi:10.2196/76218)
Supplement: Multimedia Appendix 4 [file cardio-v9-e76218-s004.docx]

# Contributing Authors

## Safer@Home

Anne Lund Krarup MD PhD Asst. Prof. (Department of Emergency Medicine and Trauma Center, Aalborg University Hospital, Aalborg, Denmark. Institute of Clinical Medicine, Aalborg University, Aalborg, Denmark);Vibe M.L. Nielsen MD PhD (Department of Emergency Medicine and Trauma Center, Aalborg University Hospital, Aalborg, Denmark. Institute of Clinical Medicine, Aalborg University, Aalborg, Denmark. Center for Prehospital and Emergency Research, Department of Clinical Medicine, Aalborg University and Aalborg University Hospital, Aalborg Denmark); Adrian Kennedy MD (Acute Assessment Unit, Airedale Hospital, Steeton, West Yorkshire, United Kingdom); Muhammad Ilyas MD (Betsi Cadwaladr University Health Board, Bangor, United Kingdom); Nikhil Mathias (Cardiff University, Cardiff, United Kingdom); Maryum Kousar (Cardiff University, Cardiff, United Kingdom); Emilie Fraisse MD (Department of Emergency Medicine, Copenhagen University Hospital – Bispebjerg and Frederiksberg, Copenhagen, Denmark); Hanne Nygaard RN MSc PhD (Department of Emergency Medicine, Copenhagen University Hospital – Bispebjerg and Frederiksberg, Copenhagen, Denmark); Camilla S. Østergård MD (Emergency Department, Holbæk Hospital, Holbæk, Denmark); Jógvan O. Matras MD (Emergency Department, Holbæk Hospital, Holbæk, Denmark); Ronja Leth MD (Emergency Department, Horsens Regional Hospital, Horsens, Denmark); Steffan Kleemeier MD (Emergency Department, Horsens Regional Hospital, Horsens, Denmark); Lone W Madsen MD PhD Assoc. Prof. (Internal Medicine, Kolding Hospital, Kolding, Denmark. Institute of Regional Research, University of Southern Denmark, Kolding, Denmark); Malene Dybdahl MD (Emergency medicine and Internal Medicine, Kolding Hospital, Kolding, Denmark); Bethan Griffith MD MPhi (Acute Medicine Department, Leighton Hospital, Mid Cheshire Hospitals NHS Foundation Trust, Cheshire, United Kingdom); Mathas Høgedal Peetz BSc (Department of Emergency Medicine, Odense University Hospital, Odense, Denmark); Julie Westergaard Andersen BSc (Department of Emergency Medicine, Odense University Hospital, Odense, Denmark); Stine H. Rasmussen, MD (Emergency Department, Regionshospitalet Randers, Randers, Denmark); Gautamananda Ray MD FRCP (Department of Acute Medicine, Royal Alexandra Hospital, Paisley, United Kingdom); Iain Keith MD MB ChB FRCP (Department of Acute Medicine, Royal Alexandra Hospital, Paisley, United Kingdom); Adnan Gebril MBchB MD (Acute Medicine, Salford Royal, Manchester, United Kingdom); Michael Dan Arvig MD PhD (Research Entity for Studies in Clinical Acute Medicine (RESCUE), Emergency Department, Slagelse Hospital, Slagelse, Denmark. Department of Clinical Medicine, University of Copenhagen, Copenhagen, Denmark. Department of Regional Health Research, University of Southern Denmark, Odense, Denmark); Emilia Passaro-Geraghty (Acute Medicine, Buckinghamshire NHS Health Trust, Stoke Mandeville Hospital, Aylesbury, United Kingdom); Jonathan P. Benhamou MD (Emergency Department, University Hospital Basel, Basel, Switzerland); Peter Biesenbach MD (Department of Emergency Medicine, University Hospital of Southern Denmark Esbjerg, Esbjerg, Denmark); Nanna G. Brix RN (Department of Emergency Medicine, University Hospital of Southern Denmark Esbjerg, Esbjerg, Denmark. Research unit for Emergency Medicine, University Hospital of Southern Denmark Esbjerg, Esbjerg, Denmark); Magda Nasher MD (Acute Medical Unit, Whiston Hospital, Mersey and West Lancashire Teaching NHS Trust, Rainhill, United Kingdom).

## Research Consortium Acute Medicine (ORCA)

Suzanne Schol-Gelok MD PhD (Emergency Department, Albert Schweitzer Hospital, Dordrecht, the Netherlands); Rianne Smit MD (Emergency Department, Albert Schweitzer Hospital, Dordrecht, the Netherlands); Sheena C. Bhagirath MD (Department of Internal Medicine, Amsterdam University Medical Center, Amsterdam, the Netherlands); Amber G. den Hollander MD (Department of Internal Medicine, Amsterdam University Medical Center, Amsterdam, the Netherlands); Wesley J. van den Busken MD (Emergency Department, Dijklander Hospital, Hoorn, the Netherlands); Tom Boeije MD (Emergency Department, Dijklander Hospital, Hoorn, the Netherlands); Marjolein NT Kremers MD PhD (Emergency Department, Erasmus University Medical Center, Rotterdam. Department of Health Services Research, and CAPHRI School for Public Health and Primary Care, Aging and Long Term Care, Maastricht, The Netherlands); Gerrie Prins MD (Department of Internal Medicine, Erasmus University Medical Center, Rotterdam, the Netherlands. Department of Intensive Care, Erasmus University Medical Center, Rotterdam, the Netherlands); Annemieke M Tinholt-van der Grift MD (Department of Internal Medicine, Gelderland Valley Hospital, Ede, the Netherlands); Saar Muller MD (Department of Internal Medicine, Gelre Hospital, Apeldoorn, the Netherlands); Prof Karin H.A.H. Kaasjager MD PhD (Julius Center, Utrecht, the Netherlands. Gelre Hospital, Apeldoorn, the Netherlands. University Medical Center Utrecht, Utrecht, the Netherlands); Eleonora M. Schipper MD (Department of Internal Medicine, Groene Hart Hospital, Gouda, the Netherlands); Barin N. Khalifa MD MSc (Department of Internal Medicine, Groene Hart Hospital, Gouda, the Netherlands); Ginette R.A. Carels MD (Department of Internal Medicine, Ikazia Hospital, Rotterdam, the Netherlands); Linda Becude MD (Division of Acute Medicine, Department of Internal Medicine, Leiden University Medical Center, Leiden, the Netherlands); Geert H Groeneveld MD PhD (Division of Acute Medicine, Department of Internal Medicine, Leiden University Medical Center, Leiden, the Netherlands); Charlotte van Noord MD PhD (Department of Internal Medicine, Maasstad Hospital, Rotterdam, the Netherlands); Arthur WE Lieveld MD (Department of Internal Medicine, Maasstad Hospital, Rotterdam, the Netherlands); Patricia M. Stassen MD PhD (Department of Internal Medicine, Division General Medicine, Section Acute Medicine, Maastricht University Medical Center, Maastricht, the Netherlands. University Maastricht, Cardiovascular Research Institute Maastricht, Maastricht, the Netherlands); Pieter W.M. de Heer MD (Department of Internal Medicine, Division General Medicine, Section Acute Medicine, Maastricht University Medical Center, Maastricht, the Netherlands); Kinge van der Heide MD (Emergency Department, Martini Hospital Groningen, Groningen, the Netherlands); Anna G.M. Rojer MD (Department of Internal Medicine, OLVG, Amsterdam, the Netherlands); Anna L.J. Verhulst MD (Department of Internal Medicine, OLVG, Amsterdam, the Netherlands); Loes M. Schepers (Department of Internal Medicine, Radboud University Medical Center, Nijmegen, the Netherlands); Koos J.H. de Leijer (Department of Internal Medicine, Radboud University Medical Center, Nijmegen, the Netherlands); Karin C.R.G.M. Daemen-Gubbels MD (Department of Internal Medicine, Tergooi Medical Center, Hilversum, the Netherlands); Roos M. Berentschot BSc (Department of Internal Medicine, Tergooi Medical Center, Hilversum, the Netherlands); Mathilde Nijkeuter MD PhD (Divison of Vital Funtions, Department of Internal Medicine, University Medical Center Utrecht, Utrecht, the Netherlands); Firas al Khoury-Hansen MD MBChB (Emergency Department, VieCuri Medical Center, Venlo, the Netherlands); Dennis Barten MD MSc (Emergency Department, VieCuri Medical Center, Venlo, the Netherlands).
